# Morpho-physiological responses and growth indices of triticale to drought and salt stresses

**DOI:** 10.1038/s41598-023-36119-y

**Published:** 2023-06-01

**Authors:** Soheyla Mohammadi Alagoz, Hashem Hadi, Mahmoud Toorchi, Tomasz Andrzej Pawłowski, Behnam Asgari Lajayer, G. W. Price, Muhammad Farooq, Tess Astatkie

**Affiliations:** 1 Department of Plant Production and Genetics, Faculty of Agriculture, Urmia University, Urmia, Iran; 2 Department of Plant Breeding and Biotechnology, Faculty of Agriculture, University of Tabriz, Tabriz, Iran; 3 Institute of Dendrology, Polish Academy of Sciences, Kórnik, Poland; 4 Faculty of Agriculture, Dalhousie University, Truro, NS B2N 5E3 Canada; 5 Department of Plant Sciences, College of Agricultural and Marine Sciences, Sultan Qaboos University, Al-Khoud 123, Muscat, Oman

**Keywords:** Physiology, Plant sciences

## Abstract

Salinity and drought are two major abiotic stresses challenging global crop production and food security. In this study, the effects of individual and combined effects of drought (at different phenological stages) and salt stresses on growth, morphology, and physiology of triticale were evaluated. For this purpose, a 3 x 4 factorial design in three blocks experiment was conducted. The stress treatments included three levels of salinity (0, 50, and 100 mM NaCl) and four levels of drought (regular irrigation as well as irrigation disruption at heading, flowering, and kernel extension stages). The stresses, individual as well as combined, caused a significant decrease in chlorophyll contents, total dry matter, leaf area index, relative water content, and grain yield of triticale. In this regard, the highest reduction was recorded under combined stresses of 100 mM NaCl and drought stress at flowering. However, an increase in soluble sugars, leaf free proline, carotenoid contents, and electrolyte leakage was noted under stress conditions compared to the control. In this regard, the highest increase in leaf free proline, soluble sugars, and carotenoid contents were noted under the combination of severe salinity and drought stress imposed at the flowering stage. Investigating the growth indices in severe salinity and water deficit stress in different phenological stages shows the predominance of ionic stress over osmotic stress under severe salinity. The highest grain yield was observed under non-saline well-watered conditions whereas the lowest grain yield was recorded under severe salinity and drought stress imposed at the flowering stage. In conclusion, the flowering stage was more sensitive than the heading and kernel extension stages in terms of water deficit. The impact of salinity and water deficit was more pronounced on soluble sugars and leaf free proline; so, these criteria can be used as physiological indicators for drought and salinity tolerance in triticale.

## Introduction

Abiotic stresses, such as heavy metals^1^, water deficit^2,3^, salinity^4,5^, temperature^6^, and flood^7^ are some of the main factors limiting crop growth and development. Among these stresses, drought, and salinity are the most critical and threaten the production of agricultural products particularly in arid and semi-arid regions, where soil nutrient and organic matter contents leading to physical instability^8^. Both stresses alter many physiological processes and morphological attributes related to plant growth and development^9,10^. Salinity and drought stresses in different plant species have been studied in most cases as separate stresses^11–14^. However, under natural conditions, crop plants face a combination of salinity and drought stresses^15^. Various researches on the response of plants to combined stresses have shown that the plant responses to a combination of stresses are unique and cannot be directly deduced from the response to each of the stresses individually^16,17^.

Photosynthetic pigments and carotenoids are critical for photosynthesis^18^ but a number of biotic and abiotic stresses adversely affect plant photosynthesis and reduce crop yields^19^. The reduction in photosynthetic pigments can be caused through the reduction of the leaf surface area, which is responsible for receiving light and achieving photosynthesis^20^. Chlorophyll and carotenoids help to neutralize singlet oxygen radicals, and their quantity can indicate plant's relative stress tolerance^21^. For instance, Ahmed et al.^22^ reported that plants grown under combined salinity and drought stress showed a significant decrease in chlorophyll and carotenoids, along with a decrease in photosynthesis, stomatal density, and transpiration rate.

Plants use osmotic regulation to cope with abiotic stresses. In this regard, plant amasses osmolytes to protect the processes inside their cells against disturbing environmental changes^23^. Proline and soluble sugars help to remove free radicals from cells and reduce the effects of stress on physiological functions by increasing the osmotic concentration inside the cell^24^. Increasing the intensity of drought and salinity stresses considerably increased leaf proline and total soluble sugars content, but this increment was more significant for salinity on wheat plants^21^. Previous studies have identified the role of proline and soluble sugars in improving tolerance against salinity and drought^25–27^.

There is an evidence indicating that sustained root development is effective in the tolerance of the plant to salinity and drought stresses^28^. Salinity stress limits main root elongation and decreases fresh and dry weight of the roots in barley genotypes^29^. Root biomass increases in dry soil compared to soils with regular irrigation^30^. The leaves determine the interception of radiation and are the main photosynthetic organs. One of the first apparent effects of salinity stress is leaf growth restriction^31^. Leaf area decreased under salinity stress in durum wheat genotypes^32^. High salt concentrations can cause a significant reduction in net assimilation rate (NAR)^33^ and leaf area index (LAI)^34^. The initial increase of LAI in wheat was associated with an increase in the number of leaves and photosynthetic leaf area at tillering. Leaf aging from the base of the stem upwards may be the reason for the decrease in leaf area index after reaching the peak. A decrease in NAR may be due to premature aging of the leaves. Under water stress conditions, the decrease in crop growth rate (CGR) may be due to a decrease in LAI and NAR. The reduction of dry matter accumulation and relative growth rate (RGR) in water deficit conditions can result from reduced CGR^35^. For instance, El-Hendawy et al.^36^ reported a significant reduction in NAR, while other studies found that drought stress caused significant reductions in LAI^37^.

Cereals are a primary source of food for human health and animal feed around the world^37^. Triticale is an annual cool-season C_3_ crop of the family Poaceae, which is an intergeneric cross between the male parent rye (*Secale* ssp.) and the female parent wheat (*Triticum* ssp.)^38^. Inclusion of triticale in crop rotation helps to reduce soil pests^39^, absorbs soil nutrients, and leads to a reduction in nutrient leaching^40^. In addition, the extensive root system of triticale leads to binding of soil particles^41^. Triticale also provides food for humans and feed for livestock, including grazed or stored forage, silage, and green fodder. The development of high-yield and stable triticale cultivars can be due to resistance to biological and abiotic stresses, which led to an increase in triticale cultivation areas throughout the world^40^.

As with other crop plants, drought and salinity stresses also pose severe threats to sustainable triticale production. Studies on the combined effects of drought and salinity stresses on triticale are lacking. Therefore, this study was conducted to investigate the effects of salinity, water deficit, and their combination on photosynthetic pigments, growth indices, and the yield of triticale. For this study, it was hypothesized that the accumulation of soluble sugars and soluble proline at different phenological stages can help to improve tolerance against abiotic stresses including drought and salinity.

## Materials and methods

The experiment was conducted in the research greenhouse, with air temperature range of 26–30 °C during the day and 17–19 °C during the night, and the relative humidity range of 57–62%, of the Faculty of Agriculture of Urmia University, located at (Lat44° 59′ 12.42″ E and long37° 39′ 24.82″ N and 1270.55 above sea level), Urmia, Iran. A factorial experiment was done in 3 blocks. The characteristics of the mixture of soil (2:1:1) used in the experiments are given in Table 1.

**Table 1:** Characteristics of the soil used in the experiment. Electrical conductivity (EC), Organic carbon (O.C), Organic Matter (O.M), Field capacity (FC), and Permanent wilting point (PWP).

	pH	EC	O.C	O.M	FC	PWP	Na^+^	K^+^	Cl^−^	Clay	Silt	Sand	Texture
		dS/m	%	%	%	%	mg kg^−1^	mg kg^−1^	mg kg^−1^	%	%	%	
Soil sample	7.46	1.28	1.96	3.38	24.06	16	122.8	281	147	32	17	51	Sandy clay loam

Seeds of triticale cultivar Giannillo-92 were obtained from Seed and Plant Improvement Institute, Karaj, Iran. The Seed and Plant Improvement Institute declared that seeds of triticale were obtained under national and international guidelines and the seeds were prepared under the supervision and permission of University of Urmia and all authors comply with all the local and national guidelines. The voucher specimens of the plants were deposited at the herbarium of Department of Horticultural Sciences, University of Urmia, and Urmia, Iran. Seeds were sown (40 seeds per pot) in pots (12 kg with a diameter of 25 × 25 cm^2^) containing a mixture of soil, sand and manure (2:1:1). All of the pots were irrigated immediately after planting. In the following stages of irrigation, the humidity of the pot was kept at the field capacity. For this purpose, holes were made in the pots, and excess water was removed from the pots through drainage. After seedling establishment, 18 plants were kept in each pot. Salinity stress levels included no salinity (control or S_0_), 50 mM NaCl (moderate salinity or S_1_), and 100 mM NaCl (severe salinity or S_2_). Salinity stress was applied from the seedling stage (5-leaf stage) and water deficit stress was applied after reaching the mentioned phenological stages by irrigation disruption at that stage. The growth stages of triticale were as germination, stem elongation, heading stage (as the stem continues to elongate, the head is pushed out of the flag leaf sheath), the flowering (begins shortly after the head has fully emerged and lasts 3–5 days, starting slightly above the middle portion of the head), and the kernel-extending stage (after the Feekes Stage Flowering is complete at the base of the spike, the remaining growth stages refer to the ripeness or maturity of the “kernel” stages). According to the growth stages of triticale, 4 levels were selected for applying water deficit stress, including regular irrigation (control or D_0_), as well as irrigation disruption (drought stress [D1]) in heading (D_1_H), flowering (D_1_F), and kernel extension (D_1_K). The experiment started on 14 July 2018 and was harvested on 03 December 2018. Flag leaf sampling was done at the end of each stage. To measure some traits including leaf free proline and photosynthetic pigments in the laboratory, the samples were kept at − 80 °C after harvesting for measuring traits.

### Leaf free proline

Leaf free proline contents were determined using the ninhydrin reaction method by Bates, et al.^42^. Frozen leaf tissues (0.2 g) were homogenized with 5 ml of 3% sulphosalicylic acid and then centrifuged at 6.000×*g* for 7 min at 25 °C. The supernatant was taken, ninhydrin reagent and glacial acetic acid were added to that. The reaction mixture was incubated at 100 °C in a water bath. The reaction mixture was extracted with 2 ml of toluene and the absorbance was measured at 520 nm against toluene as a blank using a spectrophotometer. The l-proline standard curve was used to calculate the proline content, to prepare standard proline solutions (l-Proline: Mw = 115.13 g), concentrations of 6400, 100, 80, 60, 50, 40, 30, 20, 10, and zero (µmol L^−1^) were used. The amount of absorption in plant samples was converted to proline concentration through the regression equation and was expressed as µmol g^−1^ FW.

### Soluble sugars

The soluble sugars were determined following the method of Yemm and Willis^43^. Anthrone (0.1 g) was dissolved in 45 ml of 95% (v/v) sulfuric acid to prepare the anthrone reagent. Then 50 µl of alcoholic extract + 950 µl of deionized water (final volume 1000 µl) were prepared, stored in an ice bath and 2000 µl of cold anthrone solution was added to the reaction mixture. The prepared solution was incubated for 3 min at 100 °C. After cooling, the amount of total soluble sugar was read by absorbance at 630 nm using glucose as a standard. To prepare standard solutions of soluble sugar (d-glucose: Mw = 180.16 g), concentrations of 1000, 750, 500, 250, 125 and 0 (µmol L^−1^) were used and the amount of soluble sugar was calculated using the regression equation and was expressed as mM g^−1^ DW.

### Photosynthetic pigments

To measure photosynthetic pigments, the leaf tissue was weighed in the amount of 0.1 g and homogenized with 20 ml of 80% acetone and then centrifuged at 5000*g* for 10 min at 4 °C. The absorbance of the supernatant after centrifugation was read using a spectrophotometer at 663, 645 and 470 nm. The content of chlorophyll and carotenoids were calculated based on Arnon^44^. The units were calculated based on (mg g^-1^FW), and were calculated using the following Eqs. (1–4):

1 $$\mathrm{Chlorophyll\,a }= (19.3 \times \mathrm{ A}663 - 0.86 \times \mathrm{ A}645)\mathrm{ V}/100\mathrm{ W},$$

2 $$\mathrm{Chlorophyll\,b }= (19.3 \times \mathrm{ A}645 - 3.6 \times \mathrm{ A}663)\mathrm{ V}/100\mathrm{ W},$$

3 $$\mathrm{Total\,Chlorophyll }=\mathrm{ Chlorophyll\,a }+\mathrm{ Chlorophyll\,b},$$

4 $$\mathrm{Carotenoid }= (1000\times \mathrm{A}470 - 1.82\mathrm{ Cha }- 85.02\mathrm{ Chb}) /198,$$

### Morphological traits

#### Plant height, root dry weight and grain yield

Plant height (in cm) was measured immediately after sampling by using a ruler. For measuring root dry weight (in g), root samples were placed in an oven at 75 °C for 72 h, and then their dry weight was measured. The spikes were threshed to separate grains, and grain yield (in g per plant) was recorded after air drying.

#### Growth analysis

In order to estimate the dry matter accumulation trend, two plants were randomly selected in each treatment combination and harvested from the soil surface. Then their dry weight and leaf area were determined and the mean values recorded. Sampling intervals were seven days at different stages of triticale growth (72, 79, 86, 93, 100 and 107 days after planting). To obtain dry matter weight, the samples were dried in an oven at 70 ± 5 °C for 72 h until reaching a constant weight. Leaf area was measured using a leaf area meter. Leaf area index was determined by dividing leaf area over the ground area. The total dry matter (TDM), LAI, RGR, CGR and NAR, were determined using Eqs. (5–9) ^45,46^.

5 $${\mathrm{TDM}=\mathrm{e}}^{(\mathrm{a}2+\mathrm{b}2\mathrm{t}+\mathrm{c}2{\mathrm{t}}^{2})},$$

6 $$\mathrm{LAI}={\mathrm{e}}^{(\mathrm{at}+\mathrm{b}1\mathrm{t}+\mathrm{c}1{\mathrm{t}}^{2})},$$

7 $$\mathrm{RGR}=\mathrm{b}+2\mathrm{ct}+3{\mathrm{dt}}^{2},$$

8 $$\mathrm{CGR}=\mathrm{NAR }\times \mathrm{ LAI},$$

9 $$\mathrm{NAR}=\left(\mathrm{b}2+2\mathrm{c}2\mathrm{t}\right){\mathrm{e}}^{\left(\mathrm{a}2-\mathrm{a}1\right)+\left(\mathrm{b}2-\mathrm{b}1\right)\mathrm{t}+\left(\mathrm{c}2-\mathrm{c}1\right)\mathrm{t}2},$$

where, t is the time interval of sampling and a, b, c and d are coefficients of the equations.

#### Relative water content and electrolyte leakage

Relative water contents (RWC) (%), and electrolyte leakage (EL) (%) of leaf were measured at 6 stages of triticale growth. RWC was measured using leaf discs obtained from a young leaf of each plant through the following formula^47^.

10 $$RWC=((FW-DW)/(TW-DW))\times 10,$$

where, FW is the fresh weight, DW is the dry weight, and TW is the turgid weight.

EL was calculated by^48^:

11 $$EL=L_{1}/L_{2}\times 100,$$

where, L_1_ is electric conduction of leaf after putting in the deionized water at 25 °C and L_2_ is the electric conduction of the autoclaved samples.

### Statistical analysis

The effect of Salinity (3 levels: Control, Moderate, and Severe) and Drought (4 levels: Control, D_1_F, D_1_H, and D_1_K) on morphological (plant height, root dry weight, and grain yield) and physiological (proline, soluble sugar, chlorophyll a, chlorophyll b, total chlorophyll, and carotenoid) response variables was determined using a 3 × 4 factorial design in 3 blocks. However, for LAI, TDM, RGR, CGR, NAR, RWC, and EL response variables obtained from the same design (3 × 4 factorial in 3 blocks), since their values were measured repeatedly on Day 72, 79, 86, 93, 100 and 107 after planting, Repeated Measures Analysis (RMA) was completed to determine the main and interaction effects of Salinity and Drought, and how these effects evolved during these measurement days. Akaike Information Criterion (AIC)^49^ was used to determine the most appropriate covariance structure (the covariance structure that gives AIC closest to zero) to be Compound Symmetry (CS). The analysis of both the factorial design and the RMA was completed using the Mixed Procedure of SAS^50^, and further multiple means comparison (MMC) was completed for significant (p-value < 0.05) effects by comparing the least squares means of the corresponding treatment combinations. When an interaction effect is significant, the significance of the main effects and interaction effects contained in it is ignored because doing MMC on them would give misleading results. Therefore, MMC was conducted to compare the means of the treatment combinations of the highest order significant interaction effect or the main effect(s) when there is no significant interaction effect. Letter groupings were generated using a 5% level of significance for the main effects and using a 1% level of significance for interaction effects to protect Type I experiment wise error rate from over inflation. For each response variable, the validity of model assumptions (normal distribution and constant variance assumptions on the error terms) was verified by examining the residuals as described in Montgomery^51^.

## Results

As shown in Tables 2 and 3, the analysis of variance (ANOVA) results reveal that water deficit and salinity stress had significant effects on all morphological and physiological traits, and the RMA results shown in Table 4 indicate that these effects evolve during the growing stages.

**Table 2:** ANOVA p-values that show the significance of the main and interaction effects of Salinity and Drought on morphological (Plant Height, Root Dry Weight, and Grain Yield) variables. Significant effects that require multiple means comparison are shown in bold.

Source of variation	Plant height	Root dry weight	Grain yield
Block	0.017	0.681	0.351
Salinity	**0.001**	0.001	0.001
Drought	**0.001**	0.001	0.001
Salinity × drought	0.571	**0.001**	**0.038**

**Table 3:** ANOVA p-values that show the significance of the main and interaction effects of Salinity and Drought on physiological (Proline, Soluble sugar, Chlorophyll a, Chlorophyll b, Total Chlorophyll, and Carotenoid) variables. Significant effects that require multiple means comparison are shown in bold.

Source of variation	Proline	Soluble sugar	Chlorophyll a	Chlorophyll b	Total chlorophyll	Carotenoid
Block	0.752	0.292	0.996	0.440	0.669	0.601
Salinity	0.001	0.001	0.001	0.001	0.001	0.001
Drought	0.001	0.001	0.001	0.001	0.001	0.001
Salinity × drought	**0.001**	**0.001**	**0.001**	**0.027**	**0.001**	**0.001**

**Table 4:** ANOVA p-values that show the significance of the main and interaction effects of Salinity, Drought, and Day on LAI, TDM, RGR, CGR, NAR, RWC, and EL. Significant effects that require multiple means comparison are shown in bold.

Source of variation	LAI	TDM	RGR	CGR	NAR	RWC	EL
Block	0.420	0.599	0.910	0.981	0.992	0.158	0.694
Salinity	0.001	0.001	0.001	0.103	0.066	0.001	0.001
Drought	0.001	0.001	0.535	0.504	0.424	0.001	0.001
Salinity × drought	0.950	0.121	0.625	0.667	0.644	0.348	0.001
Day	0.001	0.001	0.001	0.001	0.001	0.001	0.001
Salinity × day	**0.001**	**0.001**	**0.001**	**0.001**	**0.001**	**0.001**	0.001
Drought × day	**0.001**	**0.001**	**0.001**	**0.001**	**0.001**	**0.001**	0.001
Salinity × drought × day	0.812	0.824	0.880	0.894	0.958	0.974	**0.014**

### Proline and soluble sugars content

Salinity and drought stresses had significant interaction effect on the proline and soluble sugar content of triticale (Table 3). The combined effect of drought and severe salinity stress was more pronounced in increasing proline and soluble sugar contents (Table 5). The combined effects of salinity and drought stress also showed the highest content of proline obtained in sever salinity, drought stress at the flowering stage (S_2_D_1_F). The lowest content of proline was achieved in the control salinity and drought stress levels at the heading (S_0_D_0_H). The co-occurrence of 50 mM NaCl and drought stress (S_1_D_1_) at the heading (H), flowering (F), and kernel extension (K) stages increased the proline content (237.1%, 310.3%, and 293.3%, respectively) in comparison with the control (S_0_D_0_) of the same stages (Table 5). Also, the simultaneous application of 100 mM NaCl and water scarcity stress (S_2_D_1_) at the heading, flowering, and kernel extension stages increased proline content (316.4%, 381.0%, and 332.6%, respectively) compared to the control (S_0_D_0_) at the same stages (Table 5).

**Table 5:** Mean proline (µM g^−1^FW), soluble sugar (mM g^−1^DW), chlorophyll a, chlorophyll b, total chlorophyll, and carotenoid (mg g^−1^FW) values obtained from the 18 combinations of salinity and drought. Within each column, means sharing the same letter are not significantly different from each other.

Salinity	Drought	Proline	Soluble sugar	Chlorophyll a	Chlorophyll b	Total chlorophyll	Carotenoid
Control	D_0_F	2.53 ij	4.62 i	26.8 a	5.43 abc	32.3 ab	1.24 jk
Control	D_0_H	1.59 j	3.77 i	27.0 a	6.08 a	33.1 a	1.14 k
Control	D_0_K	2.24 ij	4.32 i	26.2 ab	5.06 bc	31.3 bc	1.04 k
Control	D_1_F	6.62 d	9.87 ef	22.1 ef	3.64 ef	25.7 f.	2.60 cd
Control	D_1_H	3.30 ghi	6.69 h	24.3 cd	5.28 bc	29.6 d	1.58 hij
Control	D_1_K	4.82 ef	7.78 gh	23.1 de	4.04 de	27.1 e	2.04 ef
Moderate	D_0_F	2.70 hij	8.83 fg	25.0 bc	5.15 bc	26.6 ef	1.32 ijk
Moderate	D_0_H	2.69 hij	4.29 i	24.7 c	5.57 ab	30.3 cd	1.20 k
Moderate	D_0_K	2.39 ij	6.91 h	21.8 fg	4.84 bc	30.1 d	1.37 h–k
Moderate	D_1_F	10.38 b	13.95 b	17.7 j	3.57 ef	21.2 i	2.87 bc
Moderate	D_1_H	5.36 de	9.83 ef	21.4 fg	4.92 bc	26.3 ef	1.94 fg
Moderate	D_1_K	8.81 c	11.65 cd	20.6 gh	3.92 e	24.6 g	2.53 cd
Severe	D_0_F	4.21 efg	12.59 bc	19.7 hi	4.87 bc	22.6 h	1.69 fgh
Severe	D_0_H	3.40 fghi	4.89 i	21.9 efg	4.76 cd	26.6 ef	1.30 ijk
Severe	D_0_K	4.01 efgh	8.46 fg	18.6 ij	4.06 de	24.6 g	1.62 ghi
Severe	D_1_F	12.17 a	16.38 a	13.5 l	1.60 g	15.1 k	3.79 a
Severe	D_1_H	6.62 d	10.64 de	18.9 ij	3.92 e	22.8 h	2.32 de
Severe	D_1_K	9.69 bc	13.07 bc	15.7 k	2.88 f.	18.6 j	3.10 b

The highest soluble sugar content was obtained in S_2_D_1_F (Table 5). In contrast, the lowest content was observed in S_0_D_0_H (Table 5). The coupling of 50 mM salinity and drought stress (S_1_D_1_) increased soluble sugar content by 160.7% at the heading stage (D_1_H), 201.9% at the flowering stage (D_1_F), and 169.7% at the kernel extension stage (D_1_K) compared with the normal condition (S_0_D_0_) of the same stages. The coexistence of 100 mM NaCl and drought stress (S_2_D_1_) enhanced the soluble sugars content by 182.2% at heading, 254.5% at the flowering and 202.5% at the kernel extension stage compared to the normal condition (S_0_D_0_) of the same stages (Table 5).

### Photosynthetic pigments

The photosynthetic pigments of leaves were significantly affected by NaCl, drought stress, and their combination (Table 3). The combination of these stresses increased the content of carotenoids (Table 5). However, chlorophyll a, b, and total chlorophyll content were reduced when salinity and water deficit stresses were applied individually or in combination (Table 5).

The highest chlorophyll a content was achieved in S_0_D_0_H, S_0_D_0_F, and S_0_D_0_K, respectively. Also, the maximum chlorophyll b (6.08 mg g^−1^ FW) and total chlorophyll (33.1 mg g^−1^ FW) were obtained in S_0_D_0_H (Table 5). The lowest content of these traits (13.5, 1.60, and 15.1 mM min^−1^, respectively) was attained in S_2_D_1_F (Table 5). On the other hand, the simultaneous application of 50 mM NaCl and water scarcity stress (S_1_D_1_) at the heading, flowering, and kernel extension stages decreased chlorophyll a (20.74%, 34.19%, and 21.37%, respectively), chlorophyll b (19.08%, 34.25%, and 22.53%, respectively) and total chlorophyll (20.54%, 34.36%, and 21.41%, respectively) compared to the control (S_0_D_0_) at the same stages (Table 5). Also, the simultaneous of severe salinity + water scarcity stress (S_2_D_1_) decreased chlorophyll a, b and total chlorophyll contents by 30.0%, 35.53%, and 31.12% at the heading, by 49.63%, 70.53% and 53.25% at the flowering and 40.08%, 43.08% and 40.58% at the kernel extension stages of, respectively in comparison with the normal condition (S_0_D_0_) of each stage (Table 5).

Based on our results the maximum carotenoid content was measured in S_2_D_1_F, (Table 5). The lowest value (1.04 and 1.20 mg g^−1^ FW) was obtained at S_0_D_0_K and S_1_D_0_H treatment, respectively (Table 5). In this regard, there was an increase of about 70.18%, 131.45%, and 143.27% in carotenoid content, under the combination of water deficit and 50 mM NaCl stress at heading (D_1_H), flowering (D_1_F) and kernel extension (D_1_K) stages in comparison with control (S_0_D_0_) at the same stages (Table 4). The interaction between severe salinity (S_2_) and drought stress (D_1_) increased carotenoid content by 103.51% at the heading, 205.65% at the flowering, and 198.08% at the kernel extension compared with the control of the same phenological stages.

### Plant height, root dry weight, and grain yield

The main effects of salinity and drought stresses on plant height were significant, but not the interaction effects (Table 2), which suggests that the differences among the salinity levels were consistent at all drought stress levels. The highest (99.7 cm) and the lowest (71.5 cm) plant heights were reached under 0 and 100 mM NaCl, respectively (Table 2). Means comparison showed that both levels of salinity stress reduced plant height by 9.13% and 28.28%, respectively (Table 6). The highest plant height was obtained in normal irrigation, and the lowest was observed in water scarcity stress at the flowering stage (Table 6). Water scarcity reduced plant height by 10.88% at the heading, 13.20% at the flowering, and 7.29% at the kernel extension stage (Table 6).

**Table 6:** Mean plant height (cm) values obtained from the three Salinity and the four Drought levels. Within each column, means sharing the same letter are not significantly different from each other.

Salinity	Plant height	Drought	Plant height
Control	99.7 a	Control	94.7 a
Moderate	90.6 b	D_1_F	82.2 c
Severe	71.5 c	D_1_H	84.4 bc
		D_1_K	87.8 b

The multiple means comparison results shown in Table 5 indicate that the highest root dry weight was obtained from no-salinity and water scarcity stress at the flowering stage (S_0_D_1_F), and the lowest amount of it was at (S_2_D_0_) (Table 7). In this regard, there was a decrease of about 56.90%, 43.79%, and 47.59% in root dry weight, under the combination of water deficit and 50 mM NaCl stress at the heading (D_1_H), the flowering (D_1_F) and the kernel extension (D_1_K) stages in comparison with their control (S_0_D_0_) and the concurrence of severe salinity and drought stress (D_1_) decreased root dry weight 73.45% at heading, 68.28% at flowering and 71.38% at kernel extension in comparison with normal condition (S_0_D_0_) (Table 7).

**Table 7:** Mean root dry weight (in g) and grain yield (in g per plant) values obtained from the 12 combinations of salinity and drought. Within each column, means sharing the same letter are not significantly different from each other.

Salinity	Drought	Root dry weight	Grain yield
Control	Control	0.290 c	2.006 a
Control	D_1_F	0.795 a	1.536 bc
Control	D_1_H	0.417 b	1.636 ab
Control	D_1_K	0.453 b	1.678 ab
Moderate	Control	0.098 d	1.245 c
Moderate	D_1_F	0.163 d	0.417 ef
Moderate	D_1_H	0.125 d	0.660 de
Moderate	D_1_K	0.152 d	0.828 d
Severe	Control	0.065 d	0.274 f
Severe	D_1_F	0.092 d	0.149 f
Severe	D_1_H	0.077 d	0.234 f
Severe	D_1_K	0.083 d	0.244 f

Salinity stress and water deficit also had a significant interaction effect on grain (crop) yield (Table 2). The multiple means comparison results showed that the yield of triticale was greatly reduced as the salinity increased (37.94 and 86.34% for S_1_ and S_2_, respectively) (Table 7). Also, our findings show that the decrease in grain yields can be due to the cessation of irrigation at any morphological stage (Table 7). The combination of salinity and drought stress demonstrates that the highest amount of grain yield was achieved under normal irrigation and no salinity (S_0_D_0_) (Table 6). The lowest yield was acquired under severe salinity and water disruption during the flowering stage of plant growth (S_2_D_1_F) (Table 7). The simultaneous 50 mM NaCl + water scarcity stress reduced the grains yield of triticale compared to normal irrigation at no salinity treatment (approximately 67.10% at the heading, 79.21% at the flowering, and 58.72%, at the kernel extension stages, respectively) (Table 7). In addition, the combination of high salinity and water deprivation stress during heading, flowering, and kernel expansion phases respectively reduced grain yields by 88.33%, 92.57%, and 87.84% compared to S_0_D_0_ (Table 7).

### Growth indices

The process of changing plant growth was evaluated using different growth indices at different phenological stages. Among the most important indicators of plant growth that can be affected by salinity and drought are total dry matter (g), LAI, RGR (g g^−1^ day^−1^), NAR (g per plant^−1^ day^−1^), and CGR (g per plant^−1^ day^−1^). The RMA results that show the significance of the main and interaction effects of salinity stress, drought and day are presented in Table 4.

#### Total dry matter and leaf area index

The multiple means comparison results of total dry matter and LAI of triticale obtained from different salinity levels measured on different days, and from water-deficit treatments measured on different days are shown in Tables 7 and 8, respectively. In all treatments, TDM and LAI increased during plant growth and reached the maximum level of 93–86 days after planting. Then at maturity (93–107 days), they showed a decreasing trend. According to the results, salinity and water deprivation stress decreased plant growth. Under the control salinity condition, the highest amount of total dry matter (4.38 g per plant) and LAI (2.90) was obtained after 93 days of planting (Table 8), and under the control droughty condition, the highest amount of total dry matter (4.46 g per plant) and LAI (3.05) was obtained after 93 days of planting (Table 9). The lowest values for total dry matter and LAI were obtained from the severe salinity and water scarcity at the flowering stage (Tables 8 and 9).

**Table 8:** Mean leaf area index (LAI), total dry matter (TDM in g), relative growth rate (RGR in g g^−1^ day^−1^), crop growth rate (CGR in g per plant^−1^ day^−1^), net assimilation rate (NAR in g per plant^−1^ day^−1^), and relative water content (RWC in %) values obtained from the 18 combinations of Salinity and Day. Within each column, means sharing the same letter are not significantly different from each other.

Salinity	Day	LAI	TDM	RGR	CGR	NAR	RWC
Control	72	1.05 n	1.22 l	0.122 c	0.148 d	0.141 ab	88.3 a
Control	79	1.84 k	2.47 j	0.074 d	0.180 bc	0.098 d	82.4 b
Control	86	2.68 c	3.95 d	0.033 e	0.131 d	0.048 e	72.6 e
Control	93	2.90 a	4.38 a	− 0.002 f	− 0.005 e	− 0.003 f	63.7 h
Control	100	2.66 d	4.23 bc	− 0.031 gh	− 0.125 fg	− 0.048 gh	55.1 j
Control	107	2.10 h	3.42 gh	− 0.053 i	− 0.180 h	− 0.084 i	50.1 k
Moderate	72	1.05 n	1.10 m	0.146 b	0.161 cd	0.153 a	82.3 b
Moderate	79	1.83 l	2.47 j	0.086 d	0.213 ab	0.116 c	75.2 d
Moderate	86	2.67 d	3.86 de	0.038 e	0.145 cd	0.054 e	67.3 g
Moderate	93	2.89 a	4.27 b	0.001 f	0.003 e	0.001 f	58.6 i
Moderate	100	2.65 e	4.18 c	− 0.024 g	− 0.100 f	− 0.038 g	50.0 k
Moderate	107	2.08 i	3.37 h	− 0.037 h	− 0.123 g	− 0.060 h	43.9 m
Severe	72	1.03 o	0.71 n	0.200 a	0.141 d	0.137 ab	77.5 c
Severe	79	1.79 m	2.13 k	0.110 c	0.235 a	0.131 bc	69.0 f
Severe	86	2.64 f	3.49 g	0.043 e	0.148 cd	0.056 e	63.0 h
Severe	93	2.87 b	3.79 e	− 0.004 f	− 0.014 e	− 0.005 f	54.6 j
Severe	100	2.61 g	3.67 f	− 0.029 gh	− 0.106 fg	− 0.040 g	45.9 l
Severe	107	2.03 j	2.79 i	− 0.032 gh	− 0.090 fg	− 0.045 gh	38.6 n

**Table 9:** Mean leaf area index (LAI), total dry matter (TDM in g), relative growth rate (RGR in g g^−1^ day^−1^), crop growth rate (CGR in g per plant^−1^ day^−1^), net assimilation rate (NAR in g per plant^−1^ day^−1^), and relative water content (RWC in %) values obtained from the 24 combinations of Drought and Day. Within each column, means sharing the same letter are not significantly different from each other.

Drought	Day	LAI	TDM	RGR	CGR	NAR	RWC
Control	72	1.05 t	1.04 m	0.166 a	0.166 bcd	0.159 a	87.5 a
Control	79	1.99 o	2.52 j	0.094 c	0.234 a	0.117 cd	80.7 c
Control	86	2.84 c	4.12 c	0.037 d	0.149 cd	0.053 e	75.1 ef
Control	93	3.05 a	4.46 a	− 0.004 e	− 0.018 e	− 0.006 f	67.0 hi
Control	100	2.82 d	4.26 b	− 0.029 fg	− 0.124 fgh	− 0.044 gh	60.6 j
Control	107	2.24 l	3.42 g	− 0.038 hij	− 0.133 gh	− 0.059 ij	53.4 l
D_1_H	72	1.04 tu	1.02 m	0.155 ab	0.151 cd	0.145 ab	81.5 c
D_1_H	79	1.76 r	2.32 k	0.091 c	0.210 ab	0.119 cd	74.0 f
D_1_H	86	2.61 h	3.66 f	0.040 d	0.148 cd	0.057 e	66.3 i
D_1_H	93	2.84 c	4.09 c	0.003 e	0.012 e	0.004 f	57.1 k
D_1_H	100	2.59 i	3.95 d	− 0.022 f	− 0.087 f	− 0.034 g	48.1 n
D_1_H	107	2.02 n	3.18 h	− 0.034 ghij	− 0.109 gh	− 0.054 hij	40.6 o
D_1_F	72	1.04 u	0.99 m	0.146 b	0.136 d	0.131 bc	78.1 d
D_1_F	79	1.65 s	2.21 l	0.088 c	0.192 abc	0.116 d	71.1 g
D_1_F	86	2.50 j	3.43 g	0.040 d	0.135 d	0.054 e	60.3 j
D_1_F	93	2.72 e	3.85 de	0.001 e	0.005 e	0.002 f	51.1 m
D_1_F	100	2.47 k	3.81 e	− 0.027 fgh	− 0.104 fg	− 0.042 ghi	41.0 o
D_1_F	107	1.90 p	2.91 i	− 0.046 ij	− 0.135 h	− 0.071 j	35.8 p
D_1_K	72	1.05 t	0.99 m	0.157 ab	0.148 cd	0.141 ab	83.8 b
D_1_K	79	1.87 q	2.37 k	0.088 c	0.203 ab	0.108 d	76.2 de
D_1_K	86	2.70 f	3.87 de	0.034 d	0.135 d	0.048 e	68.7 h
D_1_K	93	2.93 b	4.18 bc	− 0.007 e	− 0.021 e	− 0.009 f	60.6 j
D_1_K	100	2.69 g	4.09 c	− 0.033 fghi	− 0.127 fgh	− 0.048 ghi	51.8 lm
D_1_K	107	2.12 m	3.25 h	− 0.045 j	− 0.149 gh	− 0.067 j	46.9 n

#### Net assimilation rate, relative growth rate, and crop growth rate

The abiotic stresses had negative effects on the trends of NAR, RGR, and CGR (Tables 8 and 9). In all treatments, NAR and RGR decreased during plant growth and reached to a minimum level at 86–93 days after planting, then showed a negative value at maturity (93–107 days). The trend of CGR changes of triticale (Tables 8 and 9) showed that in all treatments, the CGR was low in the beginning, increased considerably thereafter up to 79 days after planting, and, then showed a declining trend at 82–107 days after planting.

#### Relative water content and electrolyte leakage

Results showed that in all of the treatment combinations, RWC decreased (Tables 8 and 9), and EL increased during plant growth under salinity and drought stress (Table 9). The highest RWC (Tables 8 and 9) and lowest EL (Table 10) were reached at S_0_D_0_ 72 days after planting. On the other hand, the lowest RWC and the highest EL were obtained on 107 days after planting (Tables 8, 9 and 10).

**Table 10:** Mean electrolyte leakage (EL in %) values obtained from the 24 combinations of Drought and Day. Means sharing the same letter are not significantly different from each other. Letters with “(2)” added (e.g., (2) t) are the second-round letters after reaching z.

Salinity	Drought	Day	EL	Salinity	Drought	Day	EL
Control	Control	72	53.3 (2)t	Moderate	D_1_F	72	62.4 (2)p
Control	Control	79	68.5 (2)o	Moderate	D_1_F	79	90.0 (2)gh
Control	Control	86	89.8 (2)hi	Moderate	D_1_F	86	113.2 tu
Control	Control	93	103.4 (2)ab	Moderate	D_1_F	93	125.2 p
Control	Control	100	111.5 uvw	Moderate	D_1_F	100	143.4 jk
Control	Control	107	138.5 l	Moderate	D_1_F	107	173.4 e
Control	D_1_H	72	54.4 (2)st	Moderate	D_1_K	72	58.6 (2)q
Control	D_1_H	79	74.4 (2)mn	Moderate	D_1_K	79	84.8 (2)j
Control	D_1_H	86	95.9 (2)de	Moderate	D_1_K	86	107.2 xyz
Control	D_1_H	93	106.7 yz	Moderate	D_1_K	93	116.9 rs
Control	D_1_H	100	116.7 s	Moderate	D_1_K	100	133.8 m
Control	D_1_H	107	139.7 l	Moderate	D_1_K	107	168.9 f.
Control	D_1_F	72	58.1 (2)qr	Severe	Control	72	62.4 (2)p
Control	D_1_F	79	87.1 (2)ij	Severe	Control	79	75.1 (2)lm
Control	D_1_F	86	109.2 wxy	Severe	Control	86	98.6 (2)cd
Control	D_1_F	93	118.2 qrs	Severe	Control	93	115.9 st
Control	D_1_F	100	129.7 no	Severe	Control	100	130.3 no
Control	D_1_F	107	144.7 ij	Severe	Control	107	186.3 d
Control	D_1_K	72	55.7 (2)rst	Severe	D_1_H	72	64.8 (2)p
Control	D_1_K	79	80.9 (2)k	Severe	D_1_H	79	81.2 (2)k
Control	D_1_K	86	101.8 (2)b	Severe	D_1_H	86	105.7 z(2)a
Control	D_1_K	93	110.2 vw	Severe	D_1_H	93	119.5 qr
Control	D_1_K	100	120.8 q	Severe	D_1_H	100	141.0 kl
Control	D_1_K	107	140.8 kl	Severe	D_1_H	107	193.7 c
Moderate	Control	72	56.6 (2)qrs	Severe	D_1_F	72	67.9 (2)o
Moderate	Control	79	71.9 (2)n	Severe	D_1_F	79	92.8 (2)fg
Moderate	Control	86	94.3 (2)ef	Severe	D_1_F	86	117.0 rs
Moderate	Control	93	109.7 vwx	Severe	D_1_F	93	131.9 mn
Moderate	Control	100	121.0 q	Severe	D_1_F	100	157.0 h
Moderate	Control	107	162.5 g	Severe	D_1_F	107	202.0 a
Moderate	D_1_H	72	57.2 (2)qrs	Severe	D_1_K	72	65.0 (2)p
Moderate	D_1_H	79	77.9 (2)l	Severe	D_1_K	79	88.5 (2)hi
Moderate	D_1_H	86	100.8 (2)bc	Severe	D_1_K	86	112.3 uv
Moderate	D_1_H	93	113.2 tu	Severe	D_1_K	93	123.8 p
Moderate	D_1_H	100	128.9 o	Severe	D_1_K	100	146.6 i
Moderate	D_1_H	107	166.7 f	Severe	D_1_K	107	197.0 b

## Discussion

### Physiological response

#### Proline and Soluble sugars

Osmoprotectants act as osmolytes and protect organisms against stress. The most essential osmolytes accumulated in plants are betaine, proline, polyols, soluble sugars and sugar alcohols. The leaf free proline and soluble sugar contents increased in response to salinity, drought and their combination and the increase was greater during the flowering stage. Biosynthesis and the buildup of osmolytes under abiotic stress conditions are responsible for ROS removal, cell redox potential balance adjustment, osmotic pressure adjustment, cell pH, protein, and membrane stabilization^52^. The accumulation of osmolytes, such as proline, soluble sugars, and protein is linked to stress tolerance^53^. Arough et al.^25^ found that in triticale, the proline content increased under salinity stress. Under drought stress conditions, an increase in proline content of barley was reported by Bandurska et al.^54^. Paul et al.^55^ demonstrated that a combination of salinity and drought stress increases the proline content of wheat genotypes. Several authors have reported increasing soluble sugar content affected by drought and salinity^56^. Leaf soluble carbohydrates are elevated as plant reserves against drought stress and decreased moisture content of soil^57^. Lynda et al.^58^ showed that the content of soluble sugar in wheat increases under salinity stress. An increase in soluble sugar content under drought stress has been reported by Mohammadkhani and Heidari^59^.

#### Photosynthetic pigments

The stress conditions caused the destruction of chloroplasts and led to the reduction of chlorophyll content. Carotenoids increased in response to osmotic stress under salinity and drought stress, which can indicate relative resistance to stress. The flowering stage is more sensitive to the combination of salinity and drought stress. Chlorophyll pigments have a fundamental role in the destruction of energy and harvesting of light under stressful conditions^60^. Reduction in chlorophyll under salinity and water deficit stress has been suggested in many plant species such as sunflower^61^, sorghum^62^. Carotenoids were similarly elevated by reducing osmotic potentials for each drought and salinity. The main act of carotenoids is to prevent the production of singlet oxygen and protect against oxidative damage^63^. Shanazari et al.^64^ reported that carotenoid content increased in wheat and triticale groups under drought stress conditions compared to control. Lim et al.^65^ showed that carotenoid contents increased under salinity stress in buckwheat compared to control, and doubled in the 50 and 100 mM NaCl. It is clear that carotenoids act as a component for photoprotection by assisting in the dispersion of extra energy.

### Yield-related

The abiotic stresses also decreased plant height and yield. Root dry weight increased with drought stress, while it decreased when salinity and drought were applied simultaneously. The effects of the combination of severe salinity and drought stress were greater in the flowering stage. Salinity reduced the fresh weight of both shoots and roots and the dry weight of roots^66^. The decrease in root growth is due to the toxic effect of high concentrations of NaCl and the imbalance in nutrient uptake by the roots^67^. Water deficit significantly increased root length, but root weight decreased significantly when salinity and drought stress occur simultaneously^68^. Increasing root weight under drought stress in rice has been reported by Toorchi et al.^69^. Plant height reduction due to drought stress may be associated with a relative decrease in swelling and water loss of the cell, which helps to reduce turgor pressure and cellular division^70^. Dugasa et al.^71^ showed that simultaneous exposure to salinity and water stress reduced the plant growth of the wheat cultivars compared to normal conditions. Ahmed et al.^72^ perceived that treating barley plants with either or a combination of salinity and drought stress showed a significant decline in plant height and root weights, with the greatest reduction in combined stress. The study by Pour-Aboughadareh et al.^73^, on durum wheat under drought stress indicated that drought stress reduced the plant height, grain yield, and biomass in all genotypes compared to control. Hafez et al.^74^ corroborated that drought stress negatively affects barley plant height. Cai and Gao^75^ reported that plant height decreased under salinity stress. Some reports have shown a salinity-induced decrease in the growth and wheat grains yield^76^, and maize^77^. According to Kheirizadeh Arough et al.^78^, drought stress reduced triticale yield. Grain yield of barley^79^ and maize^80^ is considerably affected by soil moisture constraint and change.

### Growth indices

The study of growth indices is very important in the analysis of factors effecting grain yield. They can help to determine plant growth stages by quantifying the growth and development of crop production. Growth indices are affected by salinity and drought stresses. According to the obtained results, salinity and drought stress caused a decrease in the growth indices used for this study. The results obtained in Tables 8 and 9 showed that the salinity stress of 100 mM NaCl has the greatest effect on the traits of LAI, TDM, RGR, CGR, and NAR and the flowering stage is more sensitive to drought stress compared to other growth stages. Growth indices are indicators that are used to assess plant's ability and productivity^81^. Kafi et al.^82^ stated that salt stress leads to a decrease in the water potential in the root environment, which can be the main factor in reducing the accumulation of dry matter and increasing the accumulation of solutes in plant organs. Ebrahimian and Bybordi^83^ indicated that salinity could reduce dry matter accumulating in the plant because salinity causes chlorophyll degradation and leaf discoloration and chlorosis. The study by Hajibabaee et al.^84^ on corn hybrids showed that LAI and leaf and shoot dry weight decrease under drought stress. Ihsan et al.^85^ indicated that LAI decreases in wheat under water stress. Total dry matter, LAI, RGR, and CGR were significantly affected by water stress in rapeseed^86^. Salinity stress reduced RGR, and NAR in genotypes of wheat^36^. Hirasawa et al.^87^ indicated that water deficit stress had a special effect on decreasing NAR, and water stress reduced NAR and LAI. A decrease in CGR has been reported in many studies under drought stress^88,89^. Premature aging of plant leaves leads to a further reduction in RGR^90^. Munns et al.^91^ attributed that decreasing CGR under salinity can be due to decreasing RWC, leaf area, and current photosynthesis.

### Relative water content and Electrolyte leakage

Relative water content is mentioned as a suitable indicator of the water status of the leaves, which decreases under water deficit stress and causes changes in the cell membrane and increases EL from the cells^92^. Relative water content decreases significantly under salinity stress^93^. This can be due to the reduction in water uptake^94^ and/or its harmful effect on cell wall structure^95^. Chauhan and Sanadhya^96^ indicated that individual and simultaneous occurrence of salinity and drought stresses significantly reduce RWC and increase EL in (*Brassica juncea* sp.). Studies by Mahlooji et al.^97^ on barley genotypes show that salinity stress increases EL and decreases RWC. Increasing EL may be due to the destructive effects on the plasma membrane and selective permeability. This result is similar to that obtained by^98^. A study by El-Moneim et al.^99^ on wheat genotypes, revealed that salinity and drought stress reduce RWC and leaf area.

## Conclusions

Drought and salinity stresses increased the production and accumulation of proline, soluble sugars, and carotenoids and increased electrolyte leakage. At the same time decreased chlorophyll content, growth indices, relative water content and yield of triticale. The combined effects of salinity and drought stress are greater than when each stress was applied individually. The plant's responses to stresses were different in each phenological stage. Based on the results of this study, among the phenological stages, the flowering stage was the most sensitive stage to the combination of salinity and drought stress, it was found that triticale increased the activity of its non-enzymatic defense mechanism (proline, soluble sugars, and content of carotenoid) to counteract the destructive effects of salinity and drought stress and oxidative damage caused by them.

## Data Availability

The datasets used or analyzed in the current study are available from the corresponding author upon a reasonable request.

## References

[1] Lajayer BA, Ghorbanpour M, Nikabadi S (2017). Heavy metals in contaminated environment: Destiny of secondary metabolite biosynthesis, oxidative status and phytoextraction in medicinal plants. Ecotoxicol. Environ. Saf..

[2] Heshmat K, Asgari Lajayer B, Shakiba MR, Astatkie T (2021). Assessment of physiological traits of common bean cultivars in response to water stress and molybdenum levels. J. Plant Nutr..

[3] Mohammadi Alagoz, S. *et al.* Role of root hydraulics in plant drought tolerance. *J. Plant Growth Regul.* 1–16 (2022).

[4] Aliyari Rad, S., Dehghanian, Z., Asgari Lajayer, B., Nobaharan, K. & Astatkie, T. Mitochondrial respiration and energy production under some abiotic stresses. *J. Plant Growth Regul.* 1–15 (2021).

[5] Khadem Moghadam N, Motesharezadeh B, Maali-Amiri R, Asgari Lajayer B, Astatkie T (2020). Effects of potassium and zinc on physiology and chlorophyll fluorescence of two cultivars of canola grown under salinity stress. Arab. J. Geosci..

[6] Ghassemi S, Delangiz N, Lajayer BA, Saghafi D, Maggi F (2021). Review and future prospects on the mechanisms related to cold stress resistance and tolerance in medicinal plants. Acta Ecol. Sin..

[7] Kumar S (2020). Abiotic stresses and their effects on plant growth, yield and nutritional quality of agricultural produce. Int. J. Food Sci. Agric..

[8] Alori ET, Emmanuel OC, Glick BR, Babalola OO (2020). Plant–archaea relationships: A potential means to improve crop production in arid and semi-arid regions. World J. Microbiol. Biotechnol..

[9] Ma Y, Dias MC, Freitas H (2020). Drought and salinity stress responses and microbe-induced tolerance in plants. Front. Plant Sci..

[10] Kazemi Oskuei, B. *et al.* Morphological, biochemical, and physiological responses of canola cultivars to drought stress. *Int. J. Environ. Sci. Technol.* 1–10 (2023).

[11] Obata T, Fernie AR (2012). The use of metabolomics to dissect plant responses to abiotic stresses. Cell Mol. Life Sci..

[12] Opitz N (2014). Transcriptomic complexity in young maize primary roots in response to low water potentials. BMC Genom..

[13] Hassan A (2021). Foliar application of ascorbic acid enhances salinity stress tolerance in barley (*Hordeum vulgare* L.) through modulation of morpho-physio-biochemical attributes, ions uptake, osmo-protectants and stress response genes expression. Saudi J. Biol. Sci..

[14] Alam H (2021). Negative impact of long-term exposure of salinity and drought stress on native *Tetraena mandavillei* L. Physiol. Plant.

[15] Rozema J, Flowers T (2008). Crops for a salinized world. Science.

[16] Iyer NJ, Tang Y, Mahalingam R (2013). Physiological, biochemical and molecular responses to a combination of drought and ozone in *Medicago truncatula*. Plant Cell Environ..

[17] Rollins J (2013). Leaf proteome alterations in the context of physiological and morphological responses to drought and heat stress in barley (*Hordeum vulgare* L.). J. Exp. Bot..

[18] Abdel Latef AAH, Mostofa MG, Rahman MM, Abdel-Farid IB, Tran L-SPJ (2019). Extracts from yeast and carrot roots enhance maize performance under seawater-induced salt stress by altering physio-biochemical characteristics of stressed plants. J. Plant Growth Regul..

[19] Sherin G, Aswathi KR, Puthur JT (2022). Photosynthetic functions in plants subjected to stresses are positively influenced by priming. Plant Stress.

[20] Abdel Latef AAH, Abu Alhmad MF, Kordrostami M, Abo-Baker A-BA-E, Zakir AJ (2020). Inoculation with *Azospirillum lipoferum* or *Azotobacter chroococcum* reinforces maize growth by improving physiological activities under saline conditions. J. Plant Growth Regul..

[21] Movahhedi Dehnavi M, Zarei T, Khajeeyan R, Merajipoor M (2017). Drought and salinity impacts on bread wheat in a hydroponic culture: A physiological comparison. J. Plant Physiol. Breed..

[22] Ahmed IM (2013). Difference in yield and physiological features in response to drought and salinity combined stress during anthesis in Tibetan wild and cultivated barleys. PLoS ONE.

[23] Liang X, Zhang L, Natarajan SK, Becker DF (2013). Proline mechanisms of stress survival. Antioxid. Redox Signal.

[24] Tİryakİ, İ. (2016). Drought stress and tolerance mechanisms in alfalfa (*Medicago sativa* L.). Kahramanmaraș Sütçü İmam Üniversitesi Doğa Bilimleri Dergisi.

[25] Arough YK, Sharifi RS, Sedghi M, Barmaki M (2016). Effect of zinc and bio fertilizers on antioxidant enzymes activity, chlorophyll content, soluble sugars and proline in triticale under salinity condition. Not. Bot. Hortic. Agrobot. Cluj-Napoca..

[26] Nazari Z, Seyed Sharif R, Narimani H, Mohammadi Kale Sarlou SJ (2022). Effects of water limitation, biofertilizers and nano silicon on compatible osmolytes and biochemical traits of triticale. J. Crops Improv..

[27] Sairam, R. & Tyagi, A. Physiology and molecular biology of salinity stress tolerance in plants. *Curr. Sci*. 407–421 (2004).

[28] Seo DH, Seomun S, Choi YD, Jang G (2020). Root development and stress tolerance in rice: The key to improving stress tolerance without yield penalties. Int. J. Mol. Sci..

[29] Shelden MC, Roessner U, Sharp RE, Tester M, Bacic A (2013). Genetic variation in the root growth response of barley genotypes to salinity stress. Funct. Plant Biol..

[30] dos Santos TP (2007). Effects of deficit irrigation strategies on cluster microclimate for improving fruit composition of Moscatel field-grown grapevines. Sci. Hortic..

[31] Taleisnik E (2009). Leaf expansion in grasses under salt stress. J. Plant Physiol..

[32] Chamekh, Z. *et al.* Effect of salt stress on the flag leaf area and yield components in twenty five durum wheat genotypes (*Triticum turgidum* ssp. durum). *J. New Sci.* (2014).

[33] Morales M, Sánchez-Blanco M, Olmos E, Torrecillas A, Alarcon J (1998). Changes in the growth, leaf water relations and cell ultrastructure in *Argyranthemum coronopifolium* plants under saline conditions. J. Plant Physiol..

[34] Zheng Y (2008). Higher salinity tolerance cultivars of winter wheat relieved senescence at reproductive stage. Environ. Exp. Bot..

[35] Zaman E, Karim MA, Bari MN, Akter N, Ahmed JU (2016). Growth and yield performance of selected wheat varieties under water deficit conditions. Bangladesh J. Sci. Res..

[36] El-Hendawy SE, Hu Y, Schmidhalter U (2005). Growth, ion content, gas exchange, and water relations of wheat genotypes differing in salt tolerances. Aust. J. Agric. Res..

[37] Maiti R, Satya P (2014). Research advances in major cereal crops for adaptation to abiotic stresses. GM Crops Food.

[38] Kavanagh VB, Hall LM, Hall JC (2010). Potential hybridization of genetically engineered triticale with wild and weedy relatives in Canada. Crop Sci..

[39] Cantale C (2016). Triticale for bioenergy production. J. Agric. Agric. Sci. Proc..

[40] Mergoum, M. *et al.**Cereals* 267–287 (Springer, 2009).

[41] Salmon D, Mergoum M, Gomez-Macpherson H (2004). Triticale production and management. J. Triticale Improv. Prod..

[42] Bates LS, Waldren RP, Teare I (1973). Rapid determination of free proline for water-stress studies. Plant Soil.

[43] Yemm E, Willis A (1954). The estimation of carbohydrates in plant extracts by anthrone. Biochem..

[44] Arnon DI (1949). Copper enzymes in isolated chloroplasts. Polyphenoloxidase in *Beta vulgaris*. Plant Physiol..

[45] Acquaah, G. *Principle of Crop Production, Theory, Techniques and Technology*. 460 (Prentice-Hall of India. Co. Pvt Ird, 2002).

[46] Gupta N, Gupta S (2005). Plant Physiology.

[47] Turner N (1981). Techniques and experimental approaches for the measurement of plant water status. Plant Soil.

[48] Dionisio-Sese ML, Tobita S (1998). Antioxidant responses of rice seedlings to salinity stress. J. Plant Sci..

[49] Littell RC, Henry PR, Ammerman CB (1998). Statistical analysis of repeated measures data using SAS procedures. J. Anim. Sci..

[50] Institute, I. S. *SAS 9.4 Output Delivery System: User's Guide*. (SAS Institute, 2014).

[51] Montgomery, D. C. *Design and Analysis of Experiments*, 10th ed. (Wiley, 2017).

[52] Neelapu NRR, Deepak K, Surekha C (2015). Transgenic plants for higher antioxidant, managing salt tolerance in Plants. Mol. Genet. Genom..

[53] Cheng L (2018). Changes in the physiological characteristics and baicalin biosynthesis metabolism of *Scutellaria baicalensis* Georgi under drought stress. Ind. Crops Prod.

[54] Bandurska H (2017). Regulation of proline biosynthesis and resistance to drought stress in two barley (*Hordeum vulgare* L.) genotypes of different origin. Plant Physiol. Biochem..

[55] Paul K (2019). Co-occurrence of mild salinity and drought synergistically enhances biomass and grain retardation in wheat. Front. Plant Sci..

[56] Farooq M, Hussain M, Wakeel A, Siddique KH (2015). Salt stress in maize: Effects, resistance mechanisms, and management. A review. Agron. Sustain. Dev..

[57] Hu M, Shi Z, Xu P, Li H, Zhang Z (2015). Wheat acclimate to water deficit by modifying carbohydrates metabolism, water use efficiency, and growth. Braz. J. Bot..

[58] Lynda S, Sara B, Reda DM (2016). Comparative study of the biochemical and physiological mechanisms of two varieties of durum wheat (*Triticum durum* L.) subject to salt stress. Ind. J. Sci. Technol..

[59] Mohammadkhani N, Heidari R (2008). Drought-induced accumulation of soluble sugars and proline in two maize varieties. World Appl. Sci. J..

[60] Akram NA (2018). Aminolevulinic acid and nitric oxide regulate oxidative defense and secondary metabolisms in canola (*Brassica napus* L.) under drought stress. Protoplasma.

[61] Manivannan P (2007). Growth, biochemical modifications and proline metabolism in *Helianthus annuus* L. as induced by drought stress. Colloids Surf. B.

[62] Nxele X, Klein A, Ndimba B (2017). Drought and salinity stress alters ROS accumulation, water retention, and osmolyte content in sorghum plants. S. Afr. J. Bot..

[63] Arabshahi M, Mobasser H (2017). Effect of drought stress on carotenoid and chlorophyll contents and osmolyte accumulation. Med. Chem. Res..

[64] Shanazari M, Golkar P, Mirmohammady Maibody AM (2018). Effects of drought stress on some agronomic and bio-physiological traits of *Trititicum aestivum*, Triticale, and Tritipyrum genotypes. Arch. Agron. Soil Sci..

[65] Lim J-H, Park K-J, Kim B-K, Jeong J-W, Kim H-J (2012). Effect of salinity stress on phenolic compounds and carotenoids in buckwheat (*Fagopyrum esculentum* M.) sprout. Food Chem..

[66] Gul M, Wakeel A, Steffens D, Lindberg S (2019). Potassium-induced decrease in cytosolic Na^+^ alleviates deleterious effects of salt stress on wheat (*Triticum aestivum* L.). Plant Biol..

[67] Bengough AG, McKenzie B, Hallett P, Valentine T (2011). Root elongation, water stress, and mechanical impedance: A review of limiting stresses and beneficial root tip traits. J. Exp. Bot..

[68] Ors S, Suarez DL (2017). Spinach biomass yield and physiological response to interactive salinity and water stress. Agric. Water Manag..

[69] Toorchi M, Shashidhar H, Hittalmani S, Gireesha T (2002). Rice root morphology under contrasting moisture regimes and contribution of molecular marker heterozygosity. Euphytica.

[70] Mehraban A (2019). The effects of drought stress on yield, yield components, and yield stability at different growth stages in bread wheat cultivar (*Triticum aestivum* L.). Pol. J. Environ. Stud..

[71] Dugasa MT, Cao F, Ibrahim W, Wu F (2019). Differences in physiological and biochemical characteristics in response to single and combined drought and salinity stresses between wheat genotypes differing in salt tolerance. Physiol. Plant.

[72] Ahmed IM (2013). Genotypic differences in physiological characteristics in the tolerance to drought and salinity combined stress between Tibetan wild and cultivated barley. Plant Physiol. Biochem..

[73] Pour-Aboughadareh A (2017). Physiological responses to drought stress in wild relatives of wheat: implications for wheat improvement. Acta Physiol. Plant..

[74] Hafez Y (2020). Beneficial effects of biochar and chitosan on antioxidative capacity, osmolytes accumulation, and anatomical characters of water-stressed barley plants. Agron.

[75] Cai Z-Q, Gao Q (2020). Comparative physiological and biochemical mechanisms of salt tolerance in five contrasting highland quinoa cultivars. BMC Plant Biol..

[76] Borrelli G (2011). Durum wheat salt tolerance in relation to physiological, yield and quality characters. Cereal Res. Commun..

[77] Iqbal, S., Hussain, S., Qayyaum, M. A. & Ashraf, M. *Plant Stress Physiology* (IntechOpen, 2020).

[78] Kheirizadeh Arough Y, Seyed Sharifi R, Seyed Sharifi R (2016). Bio fertilizers and zinc effects on some physiological parameters of triticale under water-limitation condition. J. Plant Interact..

[79] El-Shawy E, El-Sabagh A, Mansour M, Barutcular C (2017). A comparative study for drought tolerance and yield stability in different genotypes of barley (*Hordeum vulgare* L.). J. Exp. Biol. Agric. Sci..

[80] Sah R (2020). Impact of water deficit stress in maize: Phenology and yield components. Sci. Rep..

[81] Soleymanifard A, Pourdad S, Naseri R, Mirzaei A (2011). Effect of planting pattern on phonological characteristics and growth indices of safflower (*Carthamus tinctorius* L.) in rainfed conditions. Iran. J. Crop Sci..

[82] Kafi M, Bagheri A, Nabati J, Mehrjerdi MZ, Masomi A (2011). Effect of salinity on some physiological variables of 11 chickpea genotypes under hydroponic conditions. J. Sci. Technol. Greenh. Cult..

[83] Ebrahimian E, Bybordi A (2011). Exogenous silicium and zinc increase antioxidant enzyme activity and alleviate salt stress in leaves of sunflower. J. Food Agric. Environ..

[84] Hajibabaee M, Azizi F, Zargari K (2012). Effect of drought stress on some morphological, physiological and agronomic traits in various foliage corn hybrids. Am. Eurasian. J. Agric. Environ. Sci..

[85] Ihsan MZ, El-Nakhlawy FS, Ismail SM, Fahad S (2016). Wheat phenological development and growth studies as affected by drought and late season high temperature stress under arid environment. Front. Plant Sci..

[86] Moaveni P, Ebrahimi A, Farahani HA (2010). Studying of oil yield variations in winter rapeseed (*Brassica napus* L.) cultivars under drought stress conditions. J. Agric. Biotech. Sustain. Dev..

[87] Hirasawa T, Nakahara M, Izumi T, Iwamoto Y, Ishihara K (1998). Effects of pre-flowering soil moisture deficits on dry matter production and ecophysiological characteristics in soybean plants under well irrigated conditions during grain filling. Plant Prod. Sci..

[88] Sokoto M, Abubakar I (2015). Growth analysis of wheat (*Triticum aestivum* L.) as influenced by water stress and variety in Sokoto, Sudan Savannah, Nigeria. Agrosearch.

[89] Soleymani A (2017). Effect of drought stress on some physiological growth indices of sunflower cultivars. Environ. Stresses Crop Sci..

[90] Abdi S, Fayaz M, Chadimzade M (2007). Effect of different levels of defoliation at reproductive stage on grain yield and oil percent of two hybrid sunflower. Agric. Nat. Res. Sci. Tech..

[91] Munns R, James RA, Läuchli A (2006). Approaches to increasing the salt tolerance of wheat and other cereals. J. Exp. Bot..

[92] Fu J, Fry J, Huang B (2004). Minimum water requirements of four turfgrasses in the transition zone. HortScience.

[93] El-Tayeb M (2005). Response of barley grains to the interactive e. ect of salinity and salicylic acid. Plant Growth Regul..

[94] Parvin K (2019). Comparative physiological and biochemical changes in tomato (*Solanum lycopersicum* L.) under salt stress and recovery: role of antioxidant defense and glyoxalase systems. Antioxidants.

[95] Abdelaal KA, Hafez YM, El-Afry MM, Tantawy DS, Alshaal T (2018). Effect of some osmoregulators on photosynthesis, lipid peroxidation, antioxidative capacity, and productivity of barley (*Hordeum vulgare* L.) under water deficit stress. Environ. Sci. Pollut. Res..

[96] Chauhan R, Sanadhya D (2019). Individual and combined effect of drought and salinity on electrolyte leakage, relative leaf water content and lipid peroxidation in *Brassica juncea* sp. supplemented with salicylic acid. J. Plant Res..

[97] Mahlooji M, Sharifi RS, Razmjoo J, Sabzalian M, Sedghi M (2018). Effect of salt stress on photosynthesis and physiological parameters of three contrasting barley genotypes. Photosynthetica.

[98] El-Esawi MA (2018). Genetic variation and alleviation of salinity stress in barley (*Hordeum vulgare* L.). Molecules.

[99] El-Moneim DA, Alqahtani MM, Abdein MA, Germoush MO (2020). Drought and salinity stress response in wheat: Physiological and TaNAC gene expression analysis in contrasting Egyptian wheat genotypes. J. Plant Biotechnol..

